# MicroRNA-125a-3p, -4530, and -92a as a Potential Circulating MicroRNA Panel for Noninvasive Pancreatic Cancer Diagnosis

**DOI:** 10.1155/2022/8040419

**Published:** 2022-10-08

**Authors:** Ali Seyed Salehi, Negar Parsa-Nikoo, Farnaz Roshan-Farzad, Roshanak Shams, Mohadeseh Fathi, Hamid Asaszadeh Aghdaei, Ali Behmanesh

**Affiliations:** ^1^Basic and Molecular Epidemiology of Gastrointestinal Disorders Research Center, Research Institute for Gastroenterology and Liver Diseases, Shahid Beheshti University of Medical Sciences, Tehran, Iran; ^2^Department of Biology, Faculty of Biological Science, Islamic Azad University, North Tehran Branch, Tehran, Iran; ^3^Bone and Joint Reconstruction Research Center, Department of Orthopedics, School of Medicine, Iran University of Medical Sciences, Tehran, Iran; ^4^Department of Medical Genetics, Shahid Beheshti University of Medical Sciences, Tehran, Iran

## Abstract

MicroRNA (miRNA) expression dysregulations in pancreatic ductal adenocarcinoma (PDAC) have been studied widely for their diagnostic and prognostic utility. By the use of bioinformatics-based methods, in our previous study, we identified some potential miRNA panels for diagnosis of pancreatic cancer patients from noncancerous controls (the screening stage). In this report, we used 142 plasma samples from people with and without pancreatic cancer (PC) to conduct RT-qPCR differential expression analysis to assess the strength of the first previously proposed diagnostic panel (consisting of miR-125a-3p, miR-4530, and miR-92a-2-5p). As the result, we identified significant upregulation for all the three considered miRNAs in the serum of PC patients. After that, a three-miRNA panel in serum was developed. The area under the receiver operating characteristic curves (AUC) for the panel were 0.850, 0.910, and 0.86, respectively, indicating that it had a higher diagnostic value than individual miRNAs. Therefore, we detected a promising three-miRNA panel in the plasma for noninvasive PC diagnosis (miR-125a-3p, miR-4530, and miR-92a-2-5p).

## 1. Introduction

According to statistics from patients in England and Wales, pancreatic cancer (PC) has the lowest overall survival rate of any cancer kind, with even less than 1% overall 10-year survival and 3% overall 5-year survival [1]. In most cancers, surgical resection is the only way to cure the cancer, but most PC patients are asymptomatic until it is too late, and they may lose out on the greatest chance for a clinical procedures [2]. Existing diagnostic procedures, such as imaging technologies and tumor biopsy, nevertheless, have drawbacks, including causing further damages, being more expensive, and having lower sensitivity and specificity [3]. As a result, effective and noninvasive screening approaches are highly demanded in order to enhance overall PC survival. During the last decade, biomarker-based techniques have been proven to be effective in the early diagnosis of variety of cancers [4]. Meanwhile, the sole FDA-approved biomarker for PC diagnosis is CA19-9; however, its indiscriminate expression in other conditions such as false-positive elevation in the context of obstructive jaundice have severely hampered its practical application in PC diagnosis [5]. As a result, finding new tumor biomarkers that can provide more robust and trustworthy diagnostic information for PC detection is crucial.

MicroRNAs are a type of noncoding RNA that regulate gene expression by degrading mRNA or inhibiting translation [6]. Salmena et al. [7] suggested the competing endogenous RNA (ceRNA) hypothesis as a novel regulatory mechanism between messenger RNA (mRNA) and noncoding RNA (ncRNA). According to this theory, cross-talk occurs when ceRNAs bind to shared miRNAs in a competitive manner. Since then, many researchers have become interested in the mechanism of ceRNA [8]. In the context of cancer, tumor suppressor miRNAs and oncogenic miRNAs are two main types of miRNAs that have a role in carcinogenesis. The role of the target mRNAs in the tumor start pathway determines the classification [9–11]. Because miRNA expression profiles differ significantly across cancer types, miRNA expression profiles could well be employed as a promising noninvasive diagnostic tool [12]. Many researchers have used bioinformatics methods to identify the most potential miRNAs and genes signatures of PC through evaluating high throughput data of patients [13–15]. A rising number of research have focused on circulating miRNAs as noninvasive biomarkers for cancer diagnosis recently [16]. Circulating miRNAs can serve as noninvasive diagnostic markers for early-stage PC, as shown by two recent prospective studies that found the higher the diagnostic value of miRNAs, the closer the recruitment time to PC onset [17–19]. Numerous circulating miRNAs have been described by several researchers as putative biomarkers for PC [10, 20, 21]. In our previous study, by the use of bioinformatics-based methods, we identified some potential miRNA panels for diagnosis of pancreatic cancer patients from noncancerous controls [22]. Overall, 5 diagnostic models based on specified expression algorithms and functional features of miRNAs were proposed, each comprising of different combinations of miRNAs. In this study, by focusing on the first proposed panel consisting of miR-125a-3p, miR-4530, and miR-92a-2-5p, we aimed to analyze the expression of these miRNAs in plasma samples of 142 people with and without pancreatic cancer through RT-qPCR method. To evaluate the considered panel, two phases of validations were done. Then, we evaluated the diagnostic value of those miRNAs as a unified diagnostic panel. Ultimately, this kind of novel circulating miRNA biomarkers with high specificity and sensitivity for the early detection of PC are hoped to be applicable after more clinical evaluations.

## 2. Material and Methods

### 2.1. Ethics Statement

All study was conducted in compliance with applicable guidelines and regulations. All subjects signed an informed consent form. The Iran National Committee for Ethics in Biomedical Research approved the research (IR.SBMU.RIGLD.REC.1398.031).

### 2.2. Patients

This research included 77 histopathologically confirmed PC patients and also 65 noncancerous donors who had been referred for clinical checkup from 2017 to 2020. All participants were selected from Gastroenterology clinic, Taleghani Hospital, Shahid Beheshti University of Medical Sciences, Tehran, Iran. Prior to surgery and/or chemotherapy, blood samples were collected from subjects. Only for 47 PC Patients, other clinical characteristics such as gender, age, FBS, tobacco smoking and alcohol addiction, disease history, and CA199 level were available and documented (Supplementary Table 1). 5 ml of peripheral venous blood was taken in tubes containing EDTA and after half an hour of incubation at room temperature, centrifuged at 4000 rpm for 10 minutes. Plasma samples were then collected in 1 ml aliquots in polypropylene tubes with special lids and stored at -80° C until further biochemical analysis.

### 2.3. Study Design

As the screening and training phase of our research had been already done through comprehensive bioinformatics methods in our previous study [22], in this study, a validation phase and a final specific bioinformatics evaluation of the 3 considered miRNAs have been done. In addition, because the demographics and clinicopathological characteristics of 47 of our pancreatic cancer patients were accessible, the relationships between these variables and the expression levels of the miRNAs under consideration were also investigated.

### 2.4. Total RNA Extraction

Total RNA was isolated from 200 L of human plasma using the TRIzol reagent (Invitrogene) isolation kit (catalog number: 15596026) according to the manufacturer's protocol.

### 2.5. CDNA Synthesis and RT-qPCR

The expression analysis of miRNAs was performed using BON miR specific PCR kits. In this study, specific stem-loop primers were used to synthesize CDNA for each miRNA. The microRNA expression analysis was performed using a BON miRNA detection kit provided by STEMCELL Technology Company (catalog number: BN-0011.17). This kit uses SYBR® Green in a simple two-step PCR method to measure all desired RNAs. Briefly, the reverse transcription reaction was performed in 20 *μ*L mixture containing 4 *μ*L of RNA extracted from the plasma, 1 *μ*L BON-RT adaptor (10 *μ*M), 2 *μ*L of 10 mM dNTPs, 4 *μ*L of RT buffer, 1.0 *μ*L of AMV reverse transcriptase, and 8 *μ*L of DEPC-treated water. The tubes were incubated at 25°C for 10 minutes, 42°C for 60 minutes, and 70°C for 10 minutes for cDNA synthesis. Subsequently, the following polymerase chain reactions (PCR) were performed: 1 cycle of 95°C for 2 minutes, followed by 40 cycles of 95°C for 5 seconds, and 60°C for 30 seconds using a Corbett Research Rotor-Gene machine. The 2 − ΔΔCt approach was used to measure the relative expression levels of miRNAs to the reference miRNA (hsa-miR-24).

### 2.6. Statistical Analysis

The unpaired student *t*-test was used to compare serum miRNA expression levels in PC patients and noncancer controls. To correlate demographic and clinical characteristics between classes and their relationship with miRNA expression levels, a one-way ANOVA or chi-squared test was used. Receiver operating characteristic (ROC) curves and the area under the ROC curve (AUC) were used to assess the diagnostic performance of the detected serum miRNAs and panels. A multiple logistic model was developed using the four miRNAs. The RStudio software version 1.2.5033 for windows and GraphPad Prism 8.0 (GraphPad Software, USA) were applied for data analysis and graph drawing. A two-sided *P* value 0.05 was regarded as statistically significant.

## 3. Results

### 3.1. RT-qPCR Evaluation of Proposed miRNAs

**Figure 1:**
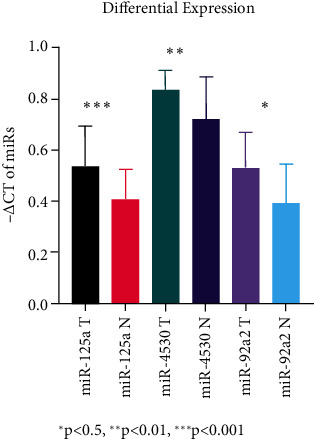
The differential expression values chart of circulating levels of miRNAs in healthy subjects and in patients with pancreatic cancer. All data was normalized to 0-1 scale.

RT-qPCR was used to assess the levels of miRNA expression in PC plasma samples vs. NCs. Interestingly, we found that miR-125a-3p, miR-4530, and miR-92a-2-5p expression levels were significantly upregulated in plasma from PC patients compared with that from in noncancerous controls (*P* < 0.05) (Table 1 and Figure 1). We also performed Pearson correlation analysis between demographics data and the expression of considered miRNAs to assess potential correlations of some clinical parameters such as gender, age, smoking, drinking, and diabetes mellitus. Except for a reverse correlation between alkaline phosphatase level with the expression level of miR-4530 (*r* = −0.416, *P* < 0.001), none of the other variables had a significant effect on serum miRNA expression in PC patients (*P* > 0.05; data not shown), demonstrating the integrity of the miRNA signatures discovered.

### 3.2. Diagnostic Performance of the 3-miRNAs Panel in Plasma

**Figure 2:**
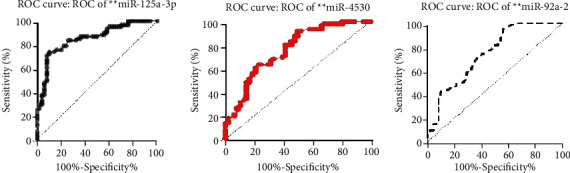
Receiver operating characteristics (ROC) curve analysis of miR-125a-3p, miR-4530, and miR-92a-2-5p in discriminating patients with pancreatic cancer from noncancerous subjects.

**Figure 3:**
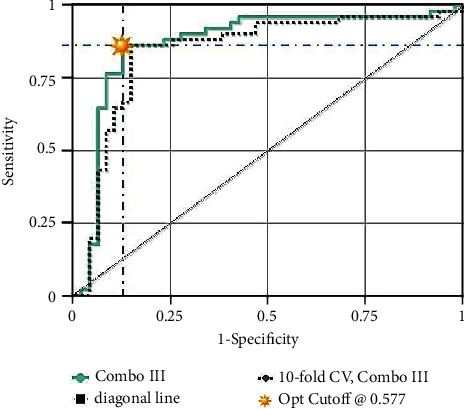
The ROC analysis of a combination of three miRNAs (miR-125a-3p, miR-4530, and miR-92a-2-5p). AUC: 0.862.

**Figure 4:**
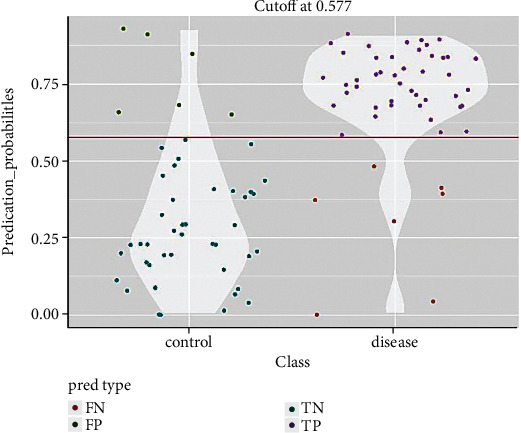
Violin plot showing the probability density of the data for the two compared classes, dependent on the previously obtained optimal cutoff on the corresponding ROC curve. FN: false negative, FP: false positive, TN: true negative, and TP: true positive.

To assess the diagnostic significance of each of the six selected miRNAs, we used the ROC analysis and computed the AUC values. The AUCs for miR-125a-3p, miR-4530, and miR-92a-2-5p in the combined cohort of the testing and validating phases were 0.853 (95% CI: 0.746-0.921; sensitivity = 86.8%, specificity = 68.8%), 0.764 (95% CI: 0.615-0.854; sensitivity = 81.5%, specificity = 64%), and 0.743 (95% CI: 0.686-0.831; sensitivity = 80%, specificity = 68.8%), respectively (Table 2 and Figure 2). Furthermore, we established a 3-miRNA panel by combining the three serum miRNAs and testing its diagnostic performance for PC comparing to a single miRNA. In a logistic regression model, the predicted probability of PC diagnosis by the panel was computed using the following formula: Logit(P) = −12.6 + 8.69 × miR − 125a + 5.7 × miR − 4530 + 8.7 × miR − 92a − 2 − 5p. The panel's ROC analysis was also performed for all testing, validating, and combining stages, and the AUCs were 0.850, 0.910, and 0.86, respectively (Figure 3).  Figure 4 demonstrates the prediction power of the combined panel in discriminating PC from controls.

### 3.3. Bioinformatics Evaluation of the Three Proposed miRNAs

**Figure 5:**
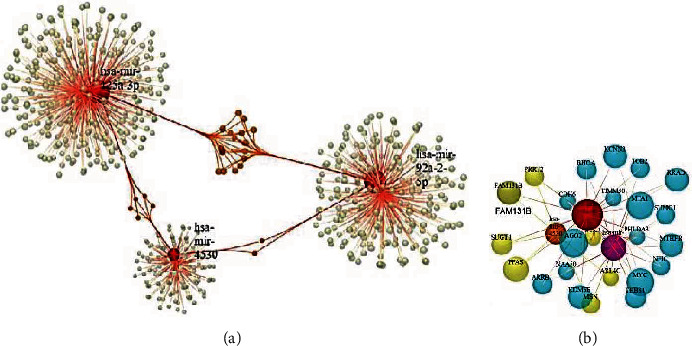
(a) Regulatory network of miRNAs and predicted target genes. Red, Green, and Orange circles represent the miRNAs, nonshared and shared target genes. (b) The extracted module demonstrating the shared targets of considered miRNAs.

Based on analytically established miRNA functions, we used miRNet (https://www.mirnet.ca/), an online miRNA pathway analysis and systems biology web-tool, to interpret the functional characteristics of the 3 miRNAs [23]. Target genes prediction was performed using miRTarbase platform for each of the specified miRNAs. A general network demonstrating all interaction between miRNAs and the target genes was prepared (Figure 5(a)) and then, amongst in common target genes of the considered miRNAs were extracted to form a final module consisting of 27 nodes and 48 edges (Figure 5(b)). Tables 3 and 4 show the results of pathway analysis utilizing the Kyoto Encyclopedia of Genes and Genomes (KEGG) and Gene Ontology (GO) analyses. mir-125a, mir-92a-2-5p, and mir-4530 were shown to have 310, 247, and 134 target genes, respectively. There were 17 target genes in common between miR-125a-3p and miR-92a-2-5p. 5 and 2 other target genes were also in common between miR-125a-3p, miR-4530, and miR-92a-2-5p. Figure 5(b) demonstrates the interactions between those genes. KEGG analysis showed that the most 3 significant pathway correlated with these genes are TGF-beta signaling pathway, colorectal cancer, and MAPK signaling pathway. GO analysis also showed that negative regulation of signal transduction, response to carbohydrate stimulus, response to drug, and notch signaling pathway were the most significant terms.

## 4. Discussion

PC has caused a serious health risk to people all around the world in recent decades, with a fatality rate approximately equal to the incidence rate [24]. To some degree, new early detection methods appear to be more effective in improving overall prognosis for PC patients than new therapy drugs [25]. The Food and Drug Administration has approved CA19.9 as the sole biomarker for clinical PC management [26, 27]. However, because CA19.9 levels rise in other malignancies and even benign conditions, it has little practical utility as a PC diagnostic biomarker [28]. A growing number of novel biomarkers or molecular targets for PC diagnosis, such as circulating miRNAs, have developed in recent years. Many miRNAs can serve as promising biomarkers for numerous diseases, including cancer, because of their stable existence in peripheral circulation [10, 29].

In our previous study, as the discovery phase, we combined several serum expression profiles of miRNAs to discover the most significant miRNA signatures helpful in PC diagnosis and then created innovative miRNA diagnosis models for PC using multiple bioinformatics methods. In the training phase, based on the area under the curve (AUC) scores, 27 differentially expressed miRNAs were selected. Afterwards, five diagnostic models comprising of distinct combinations of miRNAs, based on their significant expression algorithms and functional features, were introduced using multivariate Cox regression analysis. In this study, we focused on the first previously proposed panel to assess the expression levels and diagnostic performance of miR-125a-3p, miR4530, and miR-92a-2-5p as a panel in plasma samples from pancreatic cancer patients and noncancerous controls. In total, we analyzed 77 PC plasma samples vs. 65 NCs for the testing and validation phases. RT-qPCR was used to assess miRNA signatures. As a result, we were able to confirm that PC had significantly higher levels of miR-125a-3p, miR-4530, and miR-92a-2-5p vs. NC plasma samples. Regarding the decrease or increase of expression patterns, this discovery does not appear to be consistent with the results of our previous bioinformatics analyses, but we should take into account that the literature has published controversial statements about the expression of these miRNAs in PC or other types of malignancies. On the other hand, preliminary research suggests that expression profiles of miRNAs derived from clinical samples such as blood or tissue vary depending on the assay method (microarray, PCR, and sequencer) [30]. Aside from that, we evaluated the expression of the considered miRNAs in an Iranian population as a separate genetics pool using a different way of analysis such as RT-qPCR. In a study by Kojima et al. [30], as well as our prior bioinformatics investigations, miR-125a-3p was found to be downregulated in plasma samples of PC patients. Several other researches have also reported the downregulation of this miRNA in plasma and tumor tissues of PC patients, according to the literature [31–33]. However, our search for hsa-miR-125a-3p and pancreatic cancer in the database of Differentially Expressed miRNAs in Human Cancers (dbDEMC) [34] revealed that the expression of this miRNA was also upregulated in two other studies (GSE71533 and GSE74562). Little is known about the expression levels of miR-92a-2-5p and miR-4530 in pancreatic cancer, despite the fact that some studies have reported contradictory results regarding the expression of this genes in pancreatic cancer and also other types of cancers [30, 34–37]. Our ROC curve analyses also revealed that when used as a single marker, miR-125a-3p, miR-92a-2-5p, and miR-4530 had 85.3%, 74.3%, and 76.4% accuracies in distinguishing PC from noncancerous controls, respectively. We had hoped to improve the diagnostic performance by combining three miRNAs to test and validate one of the panels that we had previously shown [22]. We finally achieved a more robust discriminant performance with a sensitivity of 80.4%, a specificity of 87.2%, and an accuracy of 86.2% in detecting pancreatic cancer vs. healthy controls by combining three miRNAs. Even though we showed that a combo of these three serum miRNAs can be used to discriminate PC, the biological functions of those miRNAs are still being debated. To that end, we conducted another round of bioinformatics analyses to assess the functional roles of the miRNAs under consideration in interactions with their target genes. The prediction of miRNA target genes revealed that these three miRNAs share some target genes. Figure 5(a) depicts the entire network of miRNA target genes and the patterns of their interactions with one another. KEGG pathway analysis of common target genes revealed that these genes are mostly involved in TGF-beta/signaling, colorectal cancer, and MAPK signaling pathways. TGF-signaling plays a dual role in pancreatic cancer, promoting and inhibiting it depending on the stage of the disease. Some studies have shown that TGF-*β* promotes apoptosis and inhibits cell cycle progression through G1 arrest, which has a potent antiproliferative activity in early stage pancreatic cancer [38, 39]. However, some other studies have found that TGF-*β* stimulates pancreatic cancer invasion and metastasis during the advanced stages of carcinogenesis [40]. TGF-signaling therapeutic approaches have already shown usefulness in pancreatic cancer for both preclinical and clinical trials [39]. We identified three target genes (RHOA, MYC, and THBS1) implicated in this pathway which may be targeted by miR-125a-3p and miR-92a-2-5p (Table 3). Regarding pancreatic cancer tumorigenesis, studies have demonstrated that overexpression of RHOA motivates pancreatic cancer cells to invade and migrate [41]. MiRNAs have been reported to prevent the activation of human pancreatic stellate cells (hPSCs) by targeting thrombospondin 1 (THBS1) and the downstream TGF-*β* pathway, according to some studies [42]. Amongst the genes mentioned, MYC is likely the most important in the carcinogenesis of pancreatic cancer. Several studies have reported the overexpression of this gene in PC, and some resources proposed MYC-targeted therapy as an effective method for PC treatment [43, 44]. The results of GO analysis also showed that negative regulation of signal transduction, response to carbohydrate stimulus, and response to drug are the most significant terms amongst the target genes of considered miRNAs. Most of them correlated to tumorigenesis of pancreatic cancer directly or nondirectly [21, 45, 46]. Regarding the direct correlation of miR-4530 expression with serum alkaline phosphatase levels that we discovered, we should note that some studies have introduced elevated serum alkaline phosphatase levels as a marker of poor prognosis of pancreatic cancer [47, 48].

Theoretically, cancer cells are thought to leak exosomes into the circulation, which contain numerous molecular markers such as proteins, DNA, and miRNAs and transport them to distant parts of the body in order to form a new environment for future invasion [49]. The differential expression of certain miRNAs in tumor tissue samples from cancer patients should be in concordance with sera from the same patients, according to this concept; nevertheless, some research did not find this to be the case [50]. As a result, other biological pathways for the secretion of circulating miRNAs should be addressed. Our findings showed that plasma samples from PC patients had upregulation of miR-125a-3p, miR-92a-2-5p, and miR-4530, despite the fact that some researches had found downregulation of these genes (particularly for miR-125-3p) in plasma or tissues from PC patients and introduced these miRNAs with tumor suppressive role. However, the change of this miRNAs in some other studies has produced mixed results. Even if the downregulated levels of these miRNAs in plasma are linked to particular specific oncogenes involved in PC carcinogenesis, their roles in PC have been described and have to be investigated further. In this paper, we present the first evidence of a link between increased plasma levels of miR-125a-3p, miR-92a-2-5p, and miR-4530 in PC patients. Much more importantly, our findings reveal that serum miRNAs, specifically a diagnostic score based on the combination of three predictive miRNAs, may identify patients with pancreatic tumors when compared to noncancerous healthy controls. We believe that diagnosing pancreatic cancer with circulating markers in blood, which are relatively easy to obtain from most patients, is of higher quality, especially as an initial screening test. Overall, our study found new diagnostic circulating miRNAs in PC, while more researches with larger sample size is needed to confirm the diagnostic performance of the identified miRNAs in PC.

## Figures and Tables

**Table 1:** Table summarizing relevant parameters of all compared curves of 3 miRNAs.

miRNA	miR-125a-3p	miR-4530	miR-92a-2-5p
*P* value	<0.0001	0.001	<0.0001
Differences between mean ± SEM	0.23 ± 0.0241	0.186 ± 0.036	0.178 ± 0.02
AUC	0.85	0.76	0.73
Specificity	68.8	64	68.4
Sensitivity	86.8	81.5	80.1
*P* value	<0.0001	<0.0001	<0.0001

**Table 2:** Tabular view displaying performance of combined panel of miR-125a-3p, miR–92a-25p, and miR-4530.

Combinations	AUC	SE	SP	Opt cutoff
Combined cohort	0.862	0.804	0.872	0.577
Testing	0.851	0.811	0.85	0.574
Validating	0.91	0.863	0.84	0.607

**Table 3:** KEGG pathway analysis results.

Pathway	Total	Expected	Hits	*P* val
TGF-beta/signaling pathway	84	0.197	3 (RHOA, MYC, and THBS1)	0.00083
Colorectal cancer	49	0.115	2 (RHOA, MYC)	0.00556
MAPK signaling pathway	265	0.623	3 (ARRB1, MYC, and STK4)	0.0211
Leukocyte/transendothelial migration	108	0.254	2 (RHOA, MSN)	0.0253
Pathways in cancer	310	0.729	3 (RHOA, MYC, and STK4)	0.0319
Wnt signaling pathway	144	0.338	2 ((RHOA, MYC)	0.0431

**Table 4:** GO-BP term analysis results.

GO term	Total	Expected	Hits	*P* val
Negative regulation of signal transduction	790	1.27	6 (RHOA, ARRB1, MYC, THBS1, PHLDA3, and STK4)	0.00126
Response to carbohydrate stimulus	143	0.23	3 (RHOA, ARRB1, and THBS1)	0.0015
Response to drug	344	0.554	4 (RHOA, MYC, THBS1, and PFAS)	0.00204
Notch signaling pathway	177	0.285	3 (ARRB1, MYC, and AGO2)	0.00276
Cell-cell junction organization	186	0.299	3 (RHOA, CDH6, and THBS1)	0.00317
Regulation of binding	189	0.304	3 (ARRB1, THBS1, and SUMO1)	0.00332
Negative regulation of response to stimulus	967	1.56	6 (RHOA, ARRB1, MYC, THBS1, PHLDA3, and STK4)	0.00352
Positive regulation of translation	56	0.0901	2 (RHOA, THBS1)	0.00362
Negative regulation of myeloid cell differentiation	60	0.0966	2 (MYC, TOB2)	0.00415
Transforming growth factor beta receptor signaling pathway	221	0.356	3 (RHOA, MYC, and THBS1)	0.00514
Regulation of translation	228	0.367	3 (RHOA, THBS1, and AGO2)	0.00561

## Data Availability

The datasets generated during and/or analyzed during the current study are available if requested from the corresponding authors.

## Supplementary Materials

Supplementary Materials Supplementary Table 1: Demographics data of the PC patients.
