# Supplementary material for: MicroRNA-125a-3p, -4530, and -92a as a Potential Circulating MicroRNA Panel for Noninvasive Pancreatic Cancer Diagnosis
Source: Dis Markers. 2022 Oct 8;2022:8040419. doi: 10.1155/2022/8040419 (PMC9569215; doi:10.1155/2022/8040419)
Supplement: Supplementary Materials — Supplementary Table 1: Demographics data of the PC patients. [file 8040419.f1.docx]

Table 1 Supplementary table 1: Demographics data of the PC patients

| **Parameters** | | Number | opt cutoff |
| --- | --- | --- | --- |
| **Gender** | **Male** | 28 | _ |
|  | **Female** | 23 |  |
| **Smoking** | **Yes** | 16 | _ |
|  | **No** | 31 |  |
| **Jaundice** | **Yes** | 28 | _ |
|  | **No** | 19 |  |
| **BMI** | **Normal** | 22 | 18.5 -24.9 |
|  | **Overweight** | 23 |  |
|  | **Underweight** | 2 |  |
| **CA19-9** | **Normal** | 28 | 0-37 U/L |
|  | **abnormal** | 19 |  |
| **AST (SGOT)** | **Normal** | 24 | 8-45 U/L |
|  | **abnormal** | 23 |  |
| **ALT (SGPT)** | **Normal** | 25 | 7-55 U/L |
|  | **abnormal** | 22 |  |
| **Albumin** | **Normal** | 24 | 3.4 -5.4 g/dl |
|  | **abnormal** | 23 |  |
| **PLT** | **Normal** | 43 | 150000-400000 N/**µml** |
|  | **abnormal** | 4 |  |
| **FBS** | **Normal** | 40 | 80-130 mg/dl |
|  | **abnormal** | 7 |  |
| **WBC** | **Normal** | 37 | 5000-10000 N/**µl** |
|  | **abnormal** | 10 |  |
| **Lipase** | **Normal** | 39 | 0-160 U/L |
|  | **abnormal** | 8 |  |
